# 1177. Vaccinate Lurie (VaLu) a QI Project to Improve Pediatric Pre-Transplant Immunization Rates

**DOI:** 10.1093/ofid/ofab466.1370

**Published:** 2021-12-04

**Authors:** Taylor Heald-Sargent, Jordan D John, Jacquie Toia, Alexander Newman, Truc Vo, Melissa Semp, Kevin Le, Anne H Rowley, Philip Thush, Anna Joong, Natalia Panek, Ravi Jhaveri

**Affiliations:** 1 Northwestern University Feinberg School of Medicine, Ann & Robert H. Lurie Children’s Hospital, Stanley Manne Children’s Research Institute, Chicago, IL; 2 Northwestern University, Chicago, Illinois; 3 Ann & Robert H. Lurie Children’s Hospital of Chicago, Chicago, Illinois; 4 Ann and Robert H Lurie Children’s Hospital of Chicago, Chicago, IL; 5 Lurie Children’s Hospital, Chicago, Illinois; 6 Northwestern University/Lurie Children’s Hospital of Chicago, Chicago, Illinois; 7 Northwestern University/Lurie Children’s Hospital, Chicago, Illinois; 8 Northwestern University Feinberg School of Medicine; Ann & Robert H. Lurie Children’s Hospital of Chicago, Chicago, Illinois

## Abstract

**Background:**

Immunization prior to transplantation is important due to post-transplant immunosuppression. According to a national study, 15% of pediatric solid organ transplant recipients were hospitalized within 5 years post-transplant for a vaccine preventable illness or RSV. At our large academic pediatric hospital approximately 53% of heart and liver transplant recipients in 2016 -2018 were up to date with tetanus and pneumococcal vaccinations. This QI project was designed to improve our pre-transplant vaccination rates to minimize post-transplant infections.

**Methods:**

An interdisciplinary team was convened including pharmacists, nurses, nurse practitioners, and physicians from cardiology, hepatology, and infectious diseases. After evaluating our current processes and key drivers, we selected interventions to implement via the PDSA model. Our first intervention was to have team members gain access to our statewide vaccine database (ICARE). Our second cycle was to link ICARE to our electronic medical record system (EPIC) for automatic immunization record integration.

Process Map

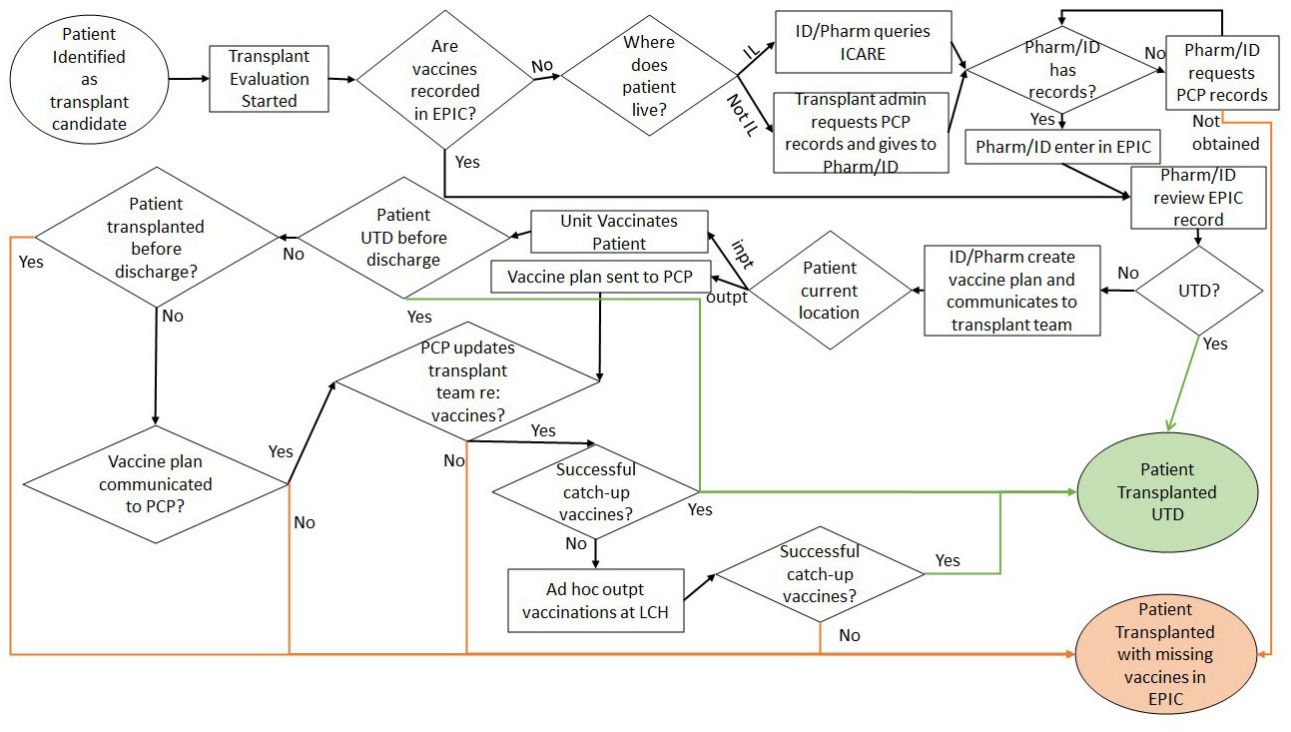

Key Driver Diagram

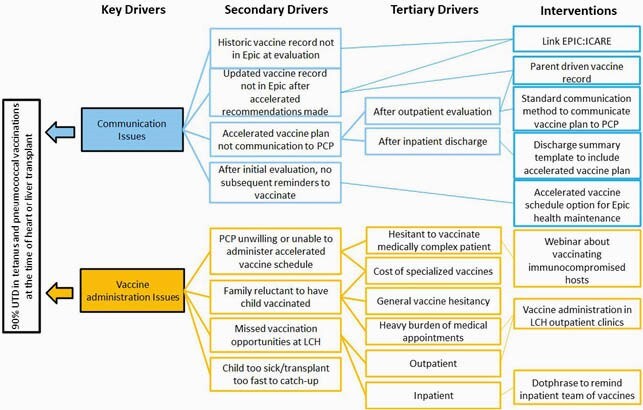

**Results:**

Our outcome measure was up to date tetanus and pneumococcal vaccines per the CDC recommendations by age at transplant, as documented in the medical record. We saw an improvement in immunization rates to 100% during the third quarter of 2020 with an overall rate of over 80% for late 2019 - mid 2020. With the understanding that our average wait time for a heart and liver transplant was 2.4 and 3.8 months, respectively, the initiation of our QI project and obtaining access to ICARE by our team members was likely related to the improved vaccination rates. Unfortunately, after the team stopped meeting during the pandemic our immunization completion rates have decreased in 2021, despite implementing institutional access to ICARE.

Control Chart

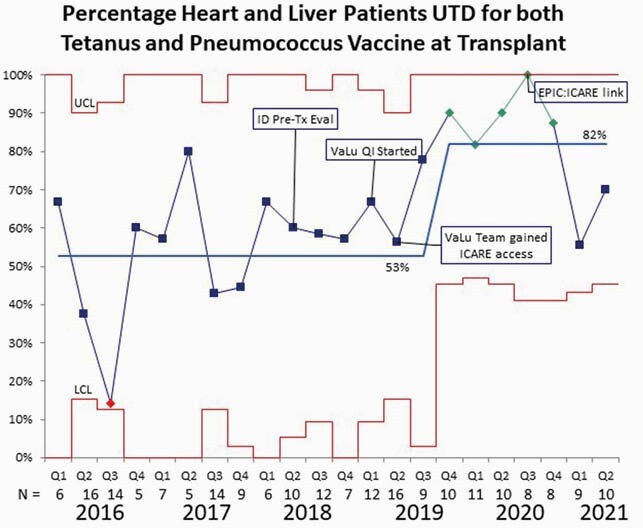

**Conclusion:**

It is possible to obtain optimal immunization rates for pneumococcal and tetanus vaccines in pediatric heart and liver transplant recipients. Our future interventions include improving vaccinations after catch-up recommendations have been made and sustaining our interventions. Additionally, we look to expand our analysis to include outcomes related to vaccine-preventable diseases after transplantation.

**Disclosures:**

**Jacquie Toia, DNP, RN, APN**, **QarTek** (Board Member) **Ravi Jhaveri, MD**, **AstraZeneca** (Consultant)**Dynavax** (Consultant)**Elsevier** (Other Financial or Material Support, Editorial Stipend as Co-editor in Chief, Clinical Therapeutics)**Seqirus** (Consultant)

